# Impacts of stress hyperglycemia ratio on functional outcomes of ischemic stroke patients treated with intravenous thrombolysis: a population-based study

**DOI:** 10.3389/fneur.2026.1685472

**Published:** 2026-03-23

**Authors:** Danhui Li, Liping Deng, Yong Han, Jimin Lai, Limin Qin, Xia Dong, Lijie Ren, Gelin Xu

**Affiliations:** 1 Department of Neurology, Shenzhen Second People's Hospital, Shenzhen, Guangdong Province, China; 2 Department of Emergency, Shenzhen Second People's Hospital, Shenzhen, Guangdong Province, China

**Keywords:** acute ischemic stroke, biomarker, functional outcome, risk stratification, stress hyperglycemia ratio, thrombolytic therapy

## Abstract

**Backgrounds:**

Stress hyperglycemia ratio (SHR) has been associated with poor outcomes in acute ischemic stroke, but the impacts of SHR on functional outcomes in stroke patients with intravenous thrombolysis remain unclear. This study aimed to investigate the predictive value of SHR on functional outcomes in stroke patients with intravenous thrombolysis.

**Methods:**

Ischemic stroke patients with intravenous thrombolysis were retrospectively enrolled in 36 centers. SHR is calculated by glucose/glycated hemoglobin. Favorable functional outcome was defined as a modified Rankin Scale score of 0–2 at 90 days after stroke onset. Multivariable logistic regression models evaluated the SHR–outcome relationship, adjusting for demographic, clinical, and laboratory parameters. Piecewise linear regression with a recursive algorithm identified potential threshold effects, and stratified analyses were performed based on key demographic and clinical factors. Sensitivity analyses were conducted in non-diabetic patients and in those with BMI < 25 kg/m^2^ and HbA1c < 6.5%.

**Results:**

A non-linear relationship was detected between SHR and functional outcomes, with an inflection point at SHR = 1.09. Below this threshold, each 0.1-unit increase in SHR was associated with a 33% increase in the odds of poor outcomes (OR = 1.33, 95% CI: 1.16–1.57, *p* < 0.001), whereas the association became non-significant above this threshold (OR = 0.91, 95% CI: 0.79–1.06, *p* = 0.201).

**Conclusion:**

SHR exhibits a threshold effect on functional outcomes in acute ischemic stroke patients receiving thrombolysis, suggesting its potential prognostic value as a risk stratification indicator in this population. These findings warrant further validation in prospective studies before clinical application.

## Introduction

Stroke is the second leading cause of death and the third leading cause of disability adjusted life-years (DALY) globally (1, 2). Ischemic stroke accounts for approximately 85% of all stroke cases, characterized by reduced cerebral blood flow due to arterial occlusion (3). The acute phase of ischemic stroke (acute ischemic stroke, AIS)—typically defined as the first hours to days after symptom onset—represents the most critical time window for therapeutic intervention and prognostic assessment. The age-standardized incidence rate is projected to increase to 89.32 per 100,000 by 2030 (4). In China, the incidence of ischemic stroke continues to increase, with recent data showing that China accounts for approximately 30% of global stroke cases, representing the highest burden worldwide (5). While intravenous thrombolysis remains the standard first-line treatment for acute ischemic stroke, its effectiveness is time-dependent, with earlier treatment associated with better functional outcomes (6). The utilization of intravenous thrombolysis has significantly improved, with recent data indicating that 22.9% of patients receive treatment within 4.5 h of symptom onset (7). Even with aggressive intravenous thrombolytic therapy, some AIS patients still experience functional deterioration and poor outcomes after thrombolysis, and the pathogenic mechanisms of functional deterioration are complex (8, 9). Currently, there are no reliable predictive indicators for the functional outcomes of stroke patients with intravenous thrombolysis in clinical practice. Therefore, screening for clinical predictive indicators is warranted for continuously improving the effectiveness of recanalization treatment in stroke patients.

The stress hyperglycemia ratio (SHR), defined as the ratio between admission glucose and estimated average glucose derived from glycated hemoglobin, has emerged as a novel clinical indicator (10). This metric accurately reflects acute stress-induced glucose elevation while accounting for chronic glycemic status. Research has demonstrated that stress hyperglycemia represents a transient elevation in blood glucose levels in response to inflammatory reactions and neuroendocrine disturbances (11). Beyond its significance in AIS, SHR has demonstrated prognostic value across various critical illnesses (12, 13).

A number of studies have examined the association between SHR and functional outcomes in AIS patients with intravenous thrombolysis, but results have been inconsistent due to small sample size and methodological flaws. While studies have indicated a positive correlation between SHR and poor outcomes, conclusions regarding optimal cutoff values and predictive performance remain controversial.

Recent studies have explored the prognostic value of SHR in acute ischemic stroke patients, but with notable limitations and inconsistencies. A systematic review and meta-analysis by Jiang et al. (11) including 11 studies (1,759 patients), found that elevated SHR was significantly associated with poor outcomes after AIS (SMD: 0.53, 95% CI: 0.35–0.71, *p* < 0.001), but with considerable heterogeneity across studies (*I*^2^ = 88%). Individual studies have reported varying effect sizes: Dai et al. (14) in 559 EVT patients found that high SHR (cut-off 1.07) predicted early neurological deterioration (OR = 4.78, 95% CI: 1.38–16.60, *p* = 0.014) and unfavorable 90-day outcomes (OR = 0.20, 95% CI: 0.08–0.50, *p* = 0.001) (14). Xiao et al. (15) observed significant associations with 90-day poor functional outcomes in 1,255 patients from two Chinese centers (15). However, these studies have critical limitations: (1) modest sample sizes (typically 200–600 patients) limiting power for detecting non-linear relationships; (2) inconsistent SHR thresholds and cut-off methodologies (ranging from 0.89 to 1.10 or using tertiles); (3) all employed linear models without systematically testing for non-linearity or threshold effects; (4) substantial heterogeneity (*I*^2^ = 88%) suggesting important unmeasured effect modifiers; and (5) conflicting results regarding predictive value in diabetic versus non-diabetic subgroups.

Given these controversies, we conducted a retrospective cohort study to evaluate the association between SHR and 90-day poor outcomes in ischemic stroke patients receiving intravenous thrombolysis. Our findings may provide a basis for future prognostic research and, pending prospective validation, could potentially inform risk stratification approaches in this patient population. Furthermore, this research contributes observational evidence that may support the development of future prognostic models for Chinese populations.

## Methods

### Study population

This retrospective cohort study was conducted across hospitals in Shenzhen, China, from 1 January 2023 to 31 December 2023. Using consecutive sampling, we enrolled 1,321 patients with acute ischemic stroke who received intravenous thrombolysis.

Patients were included if they: (1) were aged 18 years or older; (2) were diagnosed with acute ischemic stroke; and (3) received intravenous thrombolysis within 4.5 h of stroke onset. Due to the retrospective nature of this study and the heterogeneity of electronic medical record systems across participating centers, information on subsequent endovascular thrombectomy (EVT) or other interventional procedures was not systematically collected in our database. Therefore, our study findings primarily reflect outcomes in patients receiving intravenous thrombolysis as the primary reperfusion strategy. Patients were excluded if they (1) missed blood sampling for glucose and HbA1c tests before IV thrombolysis; (2) missed modified Rankin Scale (mRS) scores at 90-day follow-up. Clinical data were collected through a standardized electronic medical record system, with independent data entry by two trained researchers and verification by a third researcher to ensure data consistency.

### Ethics statement

This study protocol was approved by the Medical Ethics Committee of Shenzhen Second People’s Hospital, Shenzhen (Approval No: 2024-314-02PJ). As this was a retrospective study with anonymized patient data, the requirement for informed consent was waived in accordance with the Declaration of Helsinki and International Ethical Guidelines for Medical Research. The study strictly adhered to medical ethical principles, ensuring patient privacy and data security. All research data were stored in an encrypted database accessible only to authorized research personnel. The publication of results will strictly protect patient privacy, with no disclosure of personally identifiable information.

### Baseline assessment

The data used in this research were obtained from the Shenzhen Stroke Center Database, managed by Shenzhen Second People’s Hospital, which also serves as the designated Shenzhen Municipal Stroke Quality Control Center. The database incorporates data from 36 medical centers across Shenzhen and contains information from millions of stroke patients, aiming to establish a continuous stroke registry system to help reduce the burden of stroke in the region. To ensure data consistency across centers, the following standardization mechanisms were implemented: (1) unified data entry platform: patient data at all 36 centers are entered through a common web-based data collection interface, enforcing consistent variable definitions, data formats, and mandatory field completion; (2) standardized personnel training: data entry personnel at each center receive centralized training on uniform definitions of all clinical variables, including stroke diagnosis criteria, TOAST classification, laboratory reference ranges, and mRS assessment procedures; (3) centralized data review and quality control: the Shenzhen Stroke Center centrally collects and integrates data from all 36 centers, with regular data audits, consistency checks, and quality control meetings conducted periodically to identify and correct discrepancies; (4) geographic homogeneity: all participating centers are located within a single metropolitan area (Shenzhen), sharing broadly similar patient demographics, healthcare infrastructure, and clinical treatment protocols, reducing background center-level variation. These mechanisms substantially mitigate, though do not fully eliminate, potential inter-center data heterogeneity.

### Variables

#### Stress hyperglycemia ratio

The exposure variable, SHR, was defined as the ratio of admission blood glucose to glycated hemoglobin (13, 16). Admission blood glucose was a random (non-fasting) measurement obtained immediately upon emergency department arrival and prior to tPA administration, consistent with standard emergency stroke care practice and with prior SHR studies in acute ischemic stroke (15–17). Blood glucose was measured using the hexokinase method with a Roche Cobas 8000 automated biochemical analyzer. In the acute stroke setting, the random glucose measurement is physiologically appropriate for SHR calculation, as admission glucose primarily reflects acute neuroendocrine stress-induced elevation (mediated by catecholamine and cortisol release) rather than nutritional status, given the magnitude of stress-related glucose rise (typically 5–10 mmol/L) substantially exceeds normal postprandial fluctuation (2–3 mmol/L). HbA1c was determined by high-performance liquid chromatography (HPLC) at all 36 participating centers, following unified protocols of the Shenzhen Stroke Center Database. SHR was analyzed as both a continuous variable and a categorical variable (quartiles) to explore dose–response relationships. SHR was transformed into a categorical variable based on quartiles: Q1 (≤0.82), Q2 (>0.82 to ≤0.97), Q3 (>0.97 to ≤1.15), and Q4 (>1.15).

#### Outcome measures

The primary outcome was poor functional outcome at 90 days, defined as a modified Rankin Scale (mRS) score of 3–6. The mRS is a validated instrument ranging from 0 (no symptoms) to 6 (death), with scores of 3–6 indicating moderate to severe disability or death, representing functional dependence and poor prognosis. The 90-day mRS was assessed exclusively through standardized structured telephone interviews conducted by trained neurologists at 90 days post-stroke onset. The telephone interview followed a structured assessment protocol in which neurologists systematically inquired about each patient’s functional status and daily living activities using standardized questions corresponding to each mRS grade. Telephone-based mRS assessment has been validated as a reliable method for 90-day stroke outcome evaluation, demonstrating good agreement with face-to-face assessment (18, 19). As this was a retrospective observational study, formal prospective blinding of outcome assessors is not applicable; the 90-day mRS data were recorded in the Shenzhen Stroke Center Database as part of routine registry data collection, independently of and prior to the investigators’ analysis of the SHR-outcome relationship, thereby minimizing the risk of assessor bias. For reference, mRS scores of 0–2 are generally considered favorable outcomes, indicating functional independence (20).

#### Covariates

In our study, we selected covariates based on previous research and our clinical expertise (21–23). Covariates included (i) categorical variables: sex, education, smoking, drinking, diabetes (DM), hypertension, coronary heart disease, dyslipidemia, Stroke/TIA, atrial fibrillation (AF), antithrombotic medication use during hospitalization (antiplatelet agents and/or anticoagulants), and TOAST classification; (ii) continuous variables: age, weight, body mass index (BMI), systolic blood pressure (SBP), diastolic blood pressure (DBP), door-to-needle time (NDT), white blood cell count (WBC), high-sensitivity C-reactive protein (Hs-CRP), alanine aminotransferase (ALT), systemic hemodynamic response (SHR), glycated hemoglobin (HbA1C), arterial blood gas (ABG), low-density lipoprotein cholesterol (LDL), cystatin C (Cys-C), uric acid (UA), blood urea nitrogen (BUN), and homocysteine (HCY).

These covariates were selected based on previously reported prognostic factors and clinical expert consensus. Data collection is based on patient hospitalization cases, with laboratory information collected from electronic medical records. Admission blood glucose was measured as a random (non-fasting) sample immediately upon emergency department arrival and before tPA administration, using the hexokinase method (Roche Cobas 8000 automated biochemical analyzer). As is standard in emergency stroke care, glucose measurements were obtained regardless of fasting status. This measurement approach reflects real-world clinical practice in acute stroke management, where immediate assessment and thrombolytic treatment take priority. Other laboratory parameters (HbA1c, lipid profiles, and renal function) were measured within 24 h after admission under fasting conditions when clinically feasible. HbA1c was determined by high-performance liquid chromatography (HPLC) at all participating centers. BMI was calculated as weight in kilograms divided by height in square meters (kg/m^2^).

#### Missing data processing

In our study, multiple variables had missing data. The number of participants with missing data for WBC, BUN, LDC-C, ALT, UA, HCY, Hs-CRP, and Cys C were 8 (0.61%), 17 (1.29%), 27 (2.04%), 41 (3.10%), 48 (3.63%), 50 (3.78%), 154 (11.66%), and 177 (13.40%), respectively. To reduce bias caused by missing covariates and to maintain statistical efficiency in the target sample during the modeling process, we employed multiple imputation for the missing data. The imputation model (linear regression type, with 10 iterations) included age, sex, height, weight, BMI, WBC, BUN, FIB, LDC-C, ALT, UA, HCY, Hs-CRP, Cys C, and major treatment methods during hospitalization (including antihypertensive drugs, antiplatelet drugs, anticoagulants, and statins). The missing data analysis process used the missing at random (MAR) assumption (24).

### Statistical analysis

Continuous variables were presented as mean ± standard deviation (normal distribution) or median (interquartile range), while categorical variables were expressed as frequencies or percentages. Between-group comparisons were performed using chi-square tests (categorical variables), Student’s *t*-tests (normally distributed continuous variables), or Mann–Whitney *U* tests (skewed continuous variables). The association between stress hyperglycemia ratio and poor outcomes was analyzed in three steps: First, we constructed multilevel regression models. Model 1 was unadjusted; Model 2 adjusted for demographic characteristics only; and Model 3 further adjusted for other covariates listed in Table 1. The robustness of the results was evaluated by examining changes in effect size across different adjustment strategies. Second, we employed generalized additive models and smooth curve fitting (penalized spline method) to assess potential non-linear relationships between stress hyperglycemia ratio and poor outcomes. When non-linearity was detected, we calculated the inflection point using a recursive algorithm and constructed two piecewise linear models on both sides of the inflection point. Log-likelihood ratio tests were used to compare standard linear regression models with two-piecewise models to determine which better explained the true association. Third, we conducted stratified analyses. For continuous stratification variables, we converted them to categorical variables based on clinical cutpoints or tertiles before performing interaction tests. Effect modifications were assessed using likelihood ratio tests. For sensitivity analysis, we converted the stress hyperglycemia ratio into a categorical variable and calculated the *p*-value for trend to verify continuous variable analysis results and examine potential non-linearity. The STROBE statement was adhered to when documenting all findings (25). All analyses were performed using R statistical software.^1^ Two-sided *p*-values <0.05 were considered statistically significant. Model discrimination was assessed using the C-statistic (AUC of the receiver operating characteristic curve). Clinical utility was evaluated using decision curve analysis (DCA), which quantifies the net benefit of SHR-based risk stratification relative to ‘treat-all’ and ‘treat-none’ reference strategies across a range of threshold probabilities. These analyses are presented in Supplementary Figures S1, S2.

**Table 1:** Univariate analysis of factors associated with 90-day outcome in AIS.

Variables	Descriptive statistics	OR (95% CI)	*p*-value
Age (years)	59.31 ± 13.11	1.06 (1.04, 1.07)	<0.001^*^
Sex			
Male	928 (70.25%)	1.0 (Reference)	–
Female	393 (29.75%)	1.51 (1.10, 2.08)	0.011^*^
Education			
Illiteracy	45 (3.41%)	1.0 (Reference)	–
Primary or junior high school	763 (57.76%)	1.16 (0.51, 2.65)	0.731
High school or junior college	349 (26.42%)	0.70 (0.29, 1.68)	0.427
Bachelor’s degree or above	164 (12.41%)	0.39 (0.14, 1.07)	0.068
Smoking			
Current	368 (27.86%)	1.0 (Reference)	–
Ever	100 (7.57%)	1.68 (0.91, 3.11)	0.099
Never	853 (64.57%)	1.54 (1.06, 2.25)	0.024^*^
Drinking			
Current	173 (13.10%)	1.0 (Reference)	–
Ever	100 (7.57%)	0.89 (0.42, 1.88)	0.758
Never	998 (75.55%)	1.15 (0.72, 1.85)	0.553
Unknown	50 (3.79%)	1.06 (0.43, 2.64)	0.898
SBP (mmHg)	153.37 ± 24.08	1.00 (1.00, 1.01)	0.372
DBP (mmHg)	90.93 ± 14.94	0.99 (0.98, 1.00)	0.253
BMI (kg/m^2^)	24.06 (22.38–26.03)	1.00 (1.00, 1.00)	0.580
Hypertension			
No	587 (44.44%)	1.0 (Reference)	–
Yes	734 (55.56%)	1.11 (0.82, 1.52)	0.498
Diabetes mellitus			
No	1,095 (82.89%)	1.0 (Reference)	–
Yes	226 (17.11%)	1.33 (0.91, 1.96)	0.139
Coronary heart disease			
No	1,265 (95.76%)	1.0 (Reference)	–
Yes	56 (4.24%)	1.46 (0.74, 2.88)	0.270
Stroke/TIA			
No	1,096 (82.97%)	1.0 (Reference)	–
Yes	225 (17.03%)	1.92 (1.34, 2.75)	<0.001^*^
Atrial fibrillation			
No	1,279 (96.82%)	1.0 (Reference)	–
Yes	42 (3.18%)	2.75 (1.41, 5.40)	0.003^*^
Dyslipidemia			
No	1,286 (97.35%)	1.0 (Reference)	–
Yes	35 (2.65%)	0.54 (0.17, 1.79)	0.318
Antiplatelet therapy			
No	169 (12.97)	1.0 (Reference)	–
Yes	1,152 (87.21)	0.40(0.27,0.59)	<0.001^*^
Anticoagulants therapy			
No	1,218 (92.20)	1.0 (Reference)	–
Yes	103 (7.80)	0.92(0.51,1.65)	0.778
FBG (mmol/L)	6.64 ± 2.87	1.12 (1.07, 1.18)	<0.001^*^
HbA1c (%)	6.40 ± 1.55	1.18 (1.08, 1.28)	<0.001^*^
WBC (*10^9^/L)	8.14 ± 2.89	1.06 (1.01, 1.11)	0.021^*^
Hs-CRP (mg/L)	4.47 ± 10.21	1.03 (1.02, 1.05)	<0.001^*^
ALT (U/L)	21.00 (15.30–29.00)	0.99 (0.98, 1.00)	0.127
LDL-C (mmol/L)	3.03 ± 1.04	0.86 (0.74, 1.01)	0.073
Cys-C (mg/L)	1.20 (0.80–2.14)	0.99 (0.92, 1.06)	0.727
UA (μmol/L)	357.20 (296.00–432.85)	1.00 (1.00, 1.00)	0.066
BUN (mmol/L)	5.50 (4.40–6.89)	1.00 (0.99, 1.01)	0.851
HCY (μmol/L)	12.01 (9.69–16.17)	1.00 (0.98, 1.01)	0.567
DNT (min)	32.00 (24.00–45.00)	1.00 (1.00, 1.00)	0.751
NIHSS	4.00 (2.00–7.00)	1.16 (1.13, 1.19)	<0.001^*^
TOAST			
Large artery atherosclerosis	505 (38.29%)	1.0 (Reference)	–
Small vessel occlusion	579 (43.90%)	0.49 (0.34, 0.70)	<0.001^*^
Cardiogenic embolism	98 (7.43%)	1.46 (0.87, 2.43)	0.151
Stroke of other determined etiology	69 (5.23%)	0.76 (0.38, 1.54)	0.449
Stroke of undetermined etiology	68 (5.16%)	0.60 (0.28, 1.29)	0.192

TIA, transient ischemic attack; BMI, body mass index; SBP, systolic blood pressure; DBP, diastolic blood pressure; DNT, door-to-needle time, WBC, white blood cell; Hs-CRP, hypersensitive C-reactive protein; ALT, alanine transaminase; SHR, stress hyperglycemia ration; HbA1C, hemoglobin A1c; ABG, admission blood glucose; LDL, low density lipoprotein; Cys-C, cystatin C; UA, urine acid; BUN, blood urea nitrogen; HCY, homocysteine; TOAST, trial of org 10,172 in acute stroke treatment; NIHSS, National Institutes of Health Stroke Scale; mRS, modified Rankin scale.

Data are presented as mean ± standard deviation for continuous variables and *n* (%) for categorical variables. ^*^Indicates statistically significant association (*p* < 0.05).

### Sensitivity analysis for unmeasured confounding

To assess the robustness of our findings to potential unmeasured confounding factors (such as pre-stroke diabetes medication use, which was not systematically collected in our retrospective study), we calculated *E*-values for the primary associations identified in Model III (26). The *E*-value represents the minimum strength of association on the risk ratio scale that an unmeasured confounder would need to have with both the exposure (SHR) and the outcome (poor functional outcome), conditional on the measured covariates, to fully explain away the observed exposure–outcome association. *E*-values were calculated using the formula: *E*-value = OR + √[OR × (OR – 1)], where OR is the odds ratio from the fully adjusted model. Higher *E*-values indicate greater robustness to potential unmeasured confounding.

## Results

**Figure 1:**
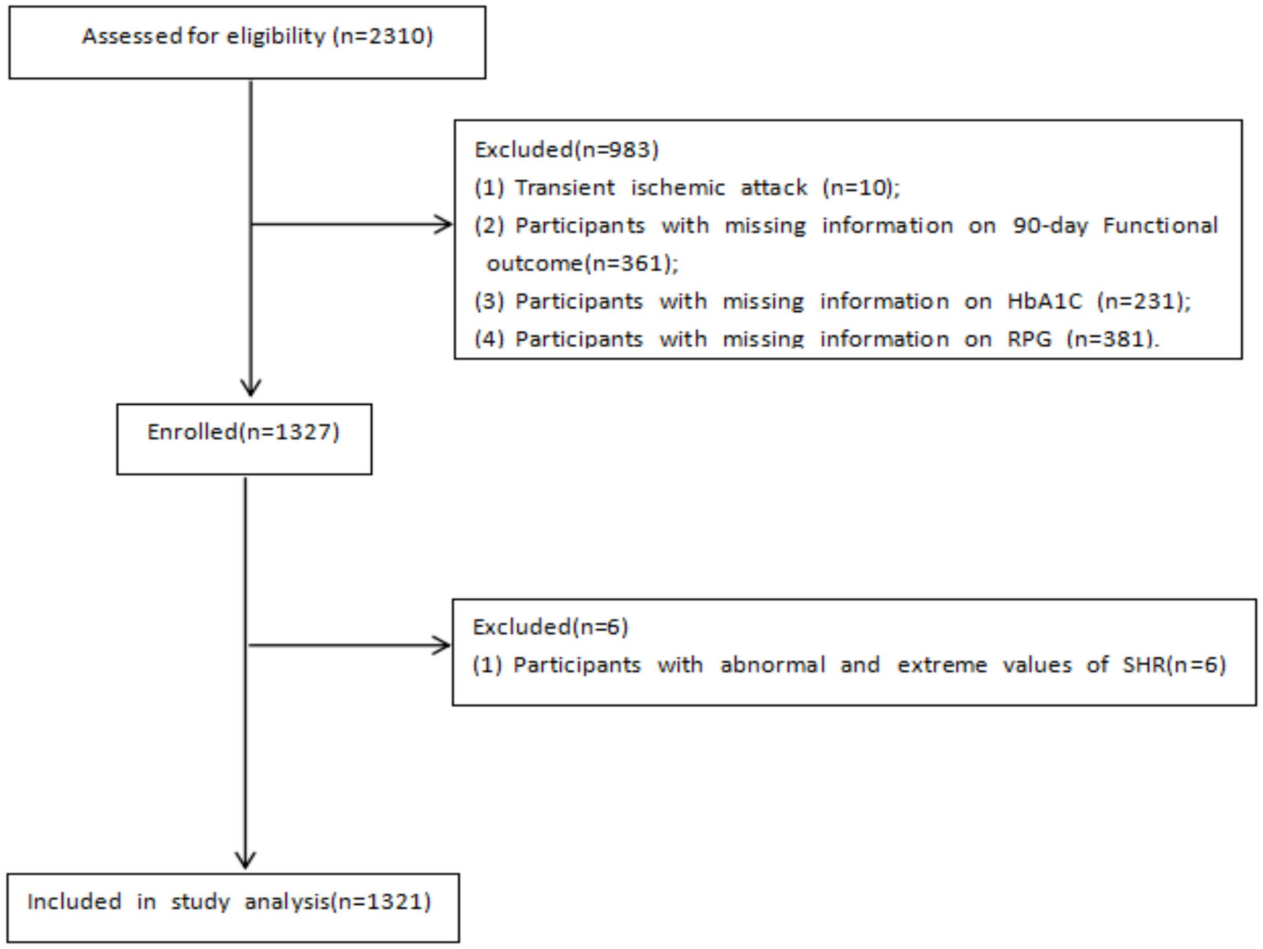
Flow chart of study sample.

A total of 1,327 patients with AIS were enrolled from the Shenzhen Stroke Center Database; of those, 6 were excluded. The inclusion and exclusion processes of the study are shown in Figure 1.

A total of 1,321 acute ischemic stroke patients were categorized based on 90-day mRS scores, with 1,129 (85.5%) having good outcomes (mRS 0–2) and 192 (14.5%) having poor outcomes (mRS 3–6). The baseline characteristics of patients (Table 2) indicate patients with poor outcomes were significantly older (66.72 ± 12.87 vs. 58.05 ± 12.74 years, *p* < 0.001), had a higher proportion of women (37.50% vs. 28.43%, *p* = 0.011), and lower educational attainment (*p* < 0.001). Medical history analysis revealed higher rates of previous stroke/TIA (26.04% vs. 15.50%, *p* < 0.001) and atrial fibrillation (6.77% vs. 2.57%, *p* = 0.002) in the poor outcome group, while diabetes, hypertension, coronary heart disease, and dyslipidemia showed no significant differences. Laboratory parameters in the poor outcome group showed elevated inflammatory markers including higher WBC (8.60 ± 2.98 vs. 8.06 ± 2.87 × 10^9/L, *p* = 0.023) and hs-CRP (8.29 ± 18.01 vs. 3.82 ± 8.01 mg/L, *p* < 0.001), higher metabolic indices including HbA1c (6.81 ± 1.86% vs. 6.33 ± 1.47%, *p* < 0.001) and admission glucose (7.70 ± 3.57 vs. 6.47 ± 2.70 mmol/L, p < 0.001), but lower ALT [19.42 (15.00–25.00) vs. 21.20 (15.60–30.00) U/L, *p* = 0.006], LDL-C (2.90 ± 0.96 vs. 3.05 ± 1.06 mmol/L, *p* = 0.043), and uric acid [342.10 (262.55–411.40) vs. 358.40 (298.75–437.70) μmol/L, *p* = 0.034]. The stress hyperglycemia ratio was higher in the poor-outcome group (1.05 ± 0.25 vs. 0.99 ± 0.26, *p* = 0.001). Initial stroke severity was greater in the poor outcome group [NIHSS: 9.00 (4.75–15.00) vs. 4.00 (2.00–6.00)]. TOAST classification differed significantly between groups (*p* < 0.001), with higher proportions of large artery atherosclerosis (48.17% vs. 36.61%) and cardiogenic embolism (12.57% vs. 6.56%) in the poor outcome group, while small vessel occlusion predominated in the good outcome group (46.28% vs. 29.84%). No significant differences were observed in BMI, blood pressure, door-to-needle time, cystatin C, BUN, or homocysteine levels.

**Table 2:** The baseline characteristics of participants (*n* = 1,321).

Characteristic	Poor outcome (mRS 3–6)	Good outcome (mRS 0–2)	*p*-value
Participants	192	1,129	
Sex			0.011
Male	120 (62.50)	808 (71.57)	
Female	72 (37.50)	321 (28.43)	
Age (years)	66.72 ± 12.87	58.05 ± 12.74	<0.001
Education			<0.001
Illiteracy	7 (3.65)	38 (3.37)	
Primary or junior high school	134 (69.79)	629 (55.71)	
High school or junior college	40 (20.83)	309 (27.37)	
Bachelor’s degree or above	11 (5.73)	153 (13.55)	
Smoking			0.060
Current	40 (20.83)	328 (29.05)	
Ever	17 (8.85)	83 (7.35)	
Never	135 (70.31)	718 (63.60)	
Drinking			0.816
Current	23 (11.98)	150 (13.29)	
Ever	12 (6.25)	88 (7.79)	
Never	150 (78.12)	848 (75.11)	
Unknown	7 (3.65)	43 (3.81)	
Diabetes	40 (20.83)	186 (16.47)	0.138
Hypertension	111 (57.81)	623 (55.18)	0.498
Coronary heart disease	11 (5.73)	45 (3.99)	0.268
Dyslipidemia	3 (1.56)	32 (2.83)	0.310
Stroke/TIA	50 (26.04)	175 (15.50)	<0.001
Atrial fibrillation	13 (6.77)	29 (2.57)	0.002
Antiplatelet therapy	147 (76.56)	1,005 (89.02)	<0.001
Anticoagulant therapy	14 (7.29)	89 (7.88)	0.778
BMI (kg/m^2^)	23.61 (21.47–25.77)	24.11 (22.49–26.06)	0.430
SBP (mmHg)	154.81 ± 24.00	153.13 ± 24.10	0.312
DBP (mmHg)	89.79 ± 16.05	91.12 ± 14.75	0.328
DNT (min)	31.00 (24.00–44.00)	32.00 (24.00–45.00)	0.553
WBC (*10^9^/L)	8.60 ± 2.98	8.06 ± 2.87	0.023
Hs-CRP (mg/L)	8.29 ± 18.01	3.82 ± 8.01	<0.001
ALT (U/L)	19.42 (15.00–25.00)	21.20 (15.60–30.00)	0.006
SHR	1.05 ± 0.25	0.99 ± 0.26	0.001
HbA1C (%)	6.81 ± 1.86	6.33 ± 1.47	<0.001
ABG (mmol/L)	7.70 ± 3.57	6.47 ± 2.70	<0.001
LDL (mmol/L)	2.90 ± 0.96	3.05 ± 1.06	0.043
Cys-C (mg/L)	1.29 (0.86–2.20)	1.20 (0.79–2.14)	0.522
UA (μmol/L)	342.10 (262.55–411.40)	358.40 (298.75–437.70)	0.034
BUN (IU/L)	5.75 (4.48–7.70)	5.45 (4.40–6.72)	0.087
HCY (μmol/L)	12.12 (9.80–16.06)	12.00 (9.60–16.20)	0.937
TOAST			<0.001
Large artery atherosclerosis	92 (48.17)	413 (36.61)	
Small vessel occlusion	57 (29.84)	522 (46.28)	
Cardiogenic embolism	24 (12.57)	74 (6.56)	
Stroke of other determined etiology	10 (5.24)	59 (5.23)	
Stroke of undetermined etiology	8 (4.19)	60 (5.32)	
NIHSS	9.00 (4.75–15.00)	4.00 (2.00–6.00)	0.107
mRS	3.59 ± 1.33	2.17 ± 1.41	<0.001

Values are mean ± standard deviation, median (quartile), or number (%).

TIA, transient ischemic attack; BMI, body mass index; SBP, systolic blood pressure; DBP, diastolic blood pressure; DNT, door-to-needle time; WBC, white blood cell; Hs-CRP, hypersensitive C-reactive protein; ALT, alanine transaminase; SHR, stress hyperglycemia ration; HbA1C, hemoglobin A1c; ABG, admission blood glucose; LDL, low density lipoprotein; Cys-C, cystatin C; UA, urine acid; BUN, blood urea nitrogen; HCY, homocysteine; TOAST, trial of org 10,172 in acute stroke treatment; NIHSS, National Institutes of Health Stroke Scale; mRS, modified Rankin scale.

### Univariate analysis of factors associated with 90-day outcome in AIS

Univariate analysis revealed that multiple factors were significantly associated with 90-day outcomes in stroke patients (Table 1). Among demographic characteristics, advanced age (OR 1.06, 95% CI 1.04–1.07, *p* < 0.001) and female (OR 1.51, 95% CI 1.10–2.08, *p* = 0.011) were associated with increased risk of poor prognosis. History of previous stroke/TIA (OR 1.92, 95% CI 1.34–2.75, *p* < 0.001) and atrial fibrillation (OR 2.75, 95% CI 1.41–5.40, *p* = 0.003) were significant predictors of unfavorable outcomes. For laboratory parameters, SHR(OR 2.35, 95% CI 1.33–4.15, *p* = 0.003), FBG (OR 1.12, 95% CI 1.07–1.18, *p* < 0.001), HbA1c (OR 1.18, 95% CI 1.08–1.28, *p* < 0.001), WBC (OR 1.06, 95% CI 1.01–1.11, *p* = 0.021), and Hs-CRP (OR 1.03, 95% CI 1.02–1.05, *p* < 0.001) levels were all associated with poor outcomes.

### Predictive values of the stress hyperglycemia ratio for functional outcomes

We evaluated the association between SHR and 90-day functional outcome using multiple logistic regression models (Table 3). After fully adjusting for demographic, clinical, and laboratory indicators (Model III), continuous SHR variable analysis showed that each 0.1-unit increase in SHR was associated with an odds ratio (OR) of 1.11 (95% CI, 1.03–1.20, *p* = 0.007) for 90-day poor functional outcomes. This indicates that SHR is an independent risk factor for 90-day functional prognosis, with a clear dose–response relationship. Quartile analysis further substantiated these findings. Compared to the reference group (Q1), patients in the third quartile (Q3) demonstrated a significantly elevated risk of functional impairment, with an adjusted OR of 2.83 (95% CI, 1.63, 4.89, *p*<0.001). Patients in the fourth quartile (Q4) also showed a significantly increased risk, with an OR of 2.08 (95% CI, 1.18, 0.67, *p* = 0.012).

**Table 3:** Relationship between SHR and 90-day functional outcome (OR per 0.1-unit increase in SHR).

Outcome	Model I (OR 95%CI)	Model II (OR 95%CI)	Model III (OR 95%CI)
SHR (per 0.1-unit)	1.09 (1.03, 1.16) 0.003	1.07 (1.01, 1.14) 0.017	1.11 (1.03, 1.20) 0.007
SHR (quartile)			
Q1	Ref	Ref	Ref
Q2	1.10 (0.67, 1.79) 0.710	1.07 (0.65, 1.76) 0.802	1.35 (0.75, 2.42) 0.320
Q3	1.90 (1.21, 2.97) 0.005	1.99 (1.26, 3.16) 0.003	2.83 (1.63, 4.89) <0.001
Q4	1.78 (1.13, 2.79) 0.012	1.64 (1.03, 2.60) 0.038	2.08 (1.18, 0.67) 0.012

Model I: we did not adjust other covariates.

Model II: we adjusted for age and sex.

Model III: we adjusted for sex, age, education, smoking, BMI, diabetes, stroke/TIA, atrial fibrillation, dyslipidemia, SBP, Hs-CRP, DNT, cystatin C, TOAST, NIHSS, pre-mRS, antiplatelet therapy, and anticoagulant therapy.

*E*-value for Model III (OR = 1.11 per 0.1-unit, equivalent to OR = 2.86 per 1-unit): 5.17 (lower CI: 2.06), indicating robustness to unmeasured confounding.

**Figure 2:**
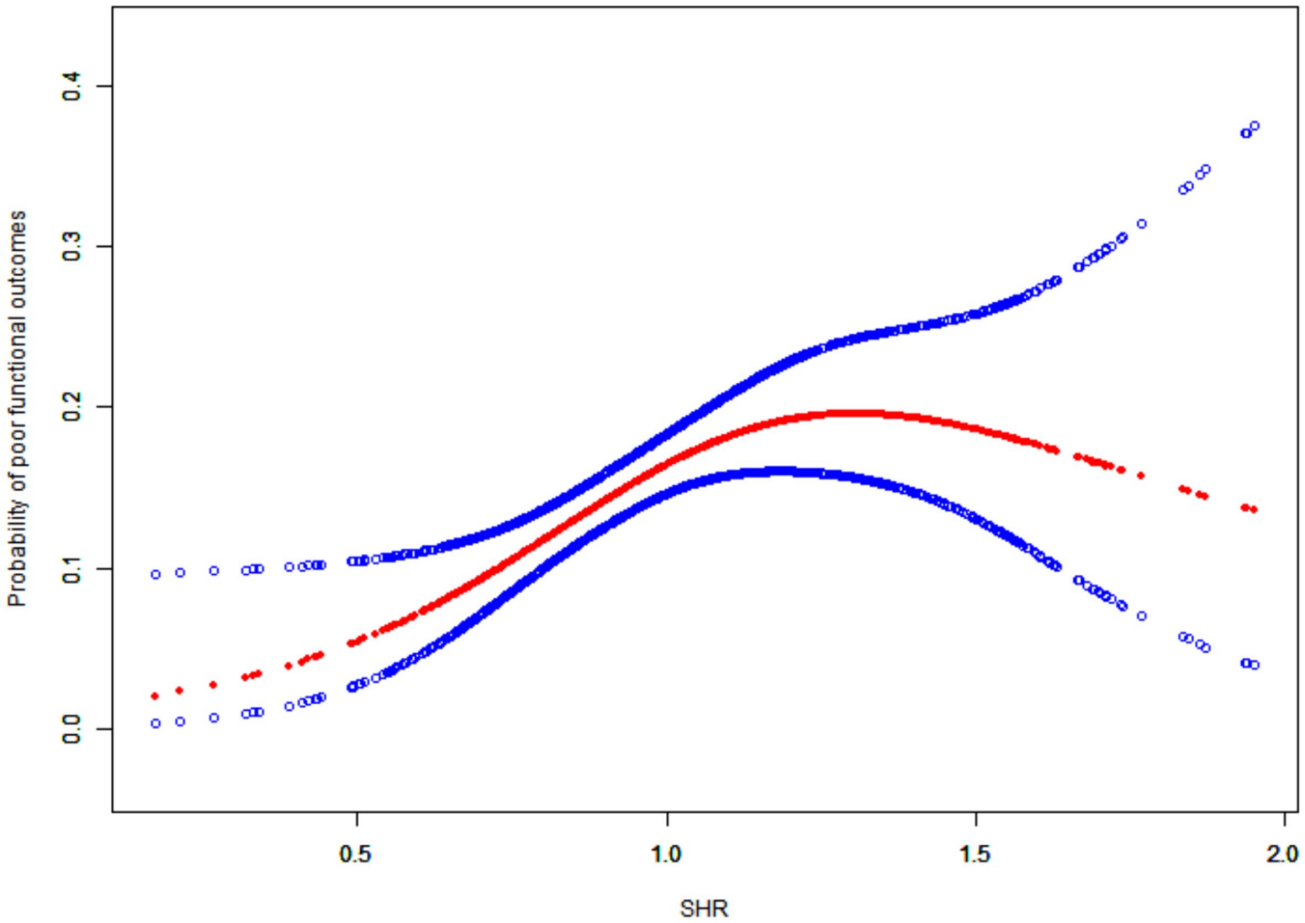
Smoothing curve fitting.

To explore the non-linear relationship between SHR and 90-day functional outcome, we performed a smooth curve fitting analysis (Figure 2). The red smooth curve represents the estimated probability of poor functional outcomes as a function of SHR, while the blue shaded area indicates the 95% confidence interval (CI). The analysis demonstrates a clear upward trend in the probability of poor functional outcomes with increasing SHR values, suggesting a significant association between elevated SHR and an adverse functional prognosis. These findings suggest an association between elevated SHR and adverse functional outcomes, which may have implications for future prognostic research in stroke patients receiving intravenous thrombolysis.

### Sensitivity analysis for unmeasured confounding

E-value analysis was conducted to assess the potential impact of unmeasured confounders on our primary findings. For the fully adjusted association between SHR (per 0.1-unit increase) and poor functional outcomes at 90 days (Model III: OR = 1.11, 95% CI: 1.03–1.20, *p* = 0.007), which is equivalent to OR = 2.86 (95% CI: 1.36–6.00, *p* = 0.006) per 1-unit increase in SHR, the E-value was 5.17, with the lower confidence limit *E*-value of 2.06. This indicates that to completely nullify the observed association, an unmeasured confounder would need to be associated with both SHR and poor functional outcomes by a risk ratio of at least 5.17-fold each, above and beyond the extensive set of measured covariates already adjusted for in our multivariable model. To provide clinical context, typical protective effects of diabetes medications on stroke outcomes range from OR 0.6–0.9 (equivalent to RR 1.1–1.7 when inverted), substantially below the threshold (RR ≥ 5.17) required to negate our observed association, suggesting strong robustness to potential unmeasured confounding from medication effects. For the threshold effect analysis (Table 4), the piecewise linear regression model identified an inflection point at SHR = 1.09. Below this threshold, each 0.1-unit increase in SHR was associated with significantly increased risk of poor outcomes (OR = 1.33, 95% CI: (1.16, 1.57), *p* < 0.001), equivalent to OR = 22.38 (95% CI: 5.18–96.73, *p* < 0.001) per 1-unit increase. This association yielded an *E*-value of 44.26 (lower confidence limit *E*-value: 9.83), indicating exceptional robustness to potential unmeasured confounding in this critical prognostic range. An unmeasured confounder would need to exhibit implausibly strong associations (RR ≥ 44.26 with both exposure and outcome) to nullify this effect, far exceeding any plausible medication effects reported in the literature. Above the inflection point (SHR > 1.09), the association was attenuated and not statistically significant (OR = 0.91 per 0.1-unit increase, 95% CI: 0.79–1.06, *p* = 0.201; equivalent to OR = 0.30 per 1-unit increase, 95% CI: 0.06–1.47, *p* = 0.138), and therefore, the *E*-value was not calculated for this segment.

**Table 4:** Threshold effect analysis of SHR and 90-day functional outcome using piece-wise linear regression (OR per 0.1-unit increase in SHR).

Outcome	Incidence of SHR OR (95%CI) per 0.1-unit increase *p*-value
Model I	
Linear effect	1.11 (1.03, 1.20) 0.007
Model II	
Inflection point (SHR)	1.09
<Inflection point	1.33 (1.16, 1.57) < 0.001
>Inflection point	0.91 (0.79, 1.06)0.201
*p* for log-likelihood ratio test	0.002

Effect: 90-day functional outcome cause: SHR.

We adjusted for sex, age, education, smoking, BMI, diabetes, stroke/TIA, atrial fibrillation, dyslipidemia, SBP, Hs-CRP, DNT, cystatin C, TOAST, NIHSS, pre-mRS, antiplatelet therapy, and anticoagulant therapy.

### Sensitivity analyses

Sensitivity analyses (Table 5) demonstrated that the first sensitivity analysis focused on participants with BMI < 25 kg/m^2^ (*n* = 844) consistently supported the initial findings, with an OR of 1.14 (95% confidence interval: 1.04–1.25, *p* = 0.006) per 0.1-unit increase in SHR, maintaining a similar pattern in quartile analysis where Q3 and Q4 groups continued to show significant associations with unfavorable outcomes. The second sensitivity analysis, concentrating on participants with HbA1c < 6.5% (*n* = 917), further validated these findings, displaying an even stronger association with an OR of 1.17 (95% confidence interval: 1.06–1.29, *p* = 0.002) per 0.1-unit increase in SHR, and quartile analysis continuing to show significant correlations, particularly in the Q3 group with an odds ratio of 4.38 (95% confidence interval: 2.09–0.15, *p* = <0.001).

**Table 5:** Relationship between SHR and 90-day functional outcome in different sensitivity analyses (OR per 0.1-unit increase in SHR).

Exposure	Model I (OR 95%CI) per 0.1-unit	Model II (OR 95%CI) per 0.1-unit
SHR	1.13 (1.03, 1.25) 0.009	1.17 (1.07, 1.30) 0.001
SHR		
Q1	Ref	Ref
Q2	1.68 (0.82, 3.43) 0.153	1.89 (0.84, 4.25) 0.125
Q3	2.86 (1.41, 5.79) 0.004	4.38 (2.09, 9.15) <0.001
Q4	2.61 (1.28, 5.34) 0.009	3.44 (1.58, 7.45) 0.002

Model I was a sensitivity analysis conducted on BMI <25 kg/m^2^ participants (*n* = 844). We adjusted for sex, age, education, smoking, diabetes, stroke/TIA, atrial fibrillation, dyslipidemia, SBP, Hs-CRP, DNT, cystatin C, TOAST, NIHSS, pre-mRS, antiplatelet therapy, and anticoagulant therapy.

Model II was a sensitivity analysis conducted on HbA1C < 6.5% participants (*n* = 917). We adjusted for sex, age, education, smoking, BMI, diabetes, stroke/TIA, atrial fibrillation, dyslipidemia, SBP, Hs-CRP, DNT, cystatin C, TOAST, NIHSS, pre-mRS, antiplatelet therapy, and anticoagulant therapy.

Odds ratios for the continuous SHR variable are reported per 0.1-unit increase. Quartile analysis represents categorical comparisons.

OR, odds ratios; CI, confidence; Ref: reference.

### Non-linear association and threshold effect analysis between SHR and 90-day functional outcome

To better characterize the relationship between SHR and 90-day functional outcome, we conducted both linear and piecewise linear regression analyses (Table 4). The results showed a significant linear effect of SHR in Model I, with an odds ratio (OR) of 1.11 (95% CI: 1.03–1.20, *p* = 0.007), indicating that each 0.1-unit increase in SHR is associated with an 11% increased risk of poor functional outcomes. Further analysis revealed an inflection point at SHR = 1.09 in Model II (log-likelihood ratio test, *p* = 0.002). Below this threshold, each 0.1-unit increase in SHR was associated with significantly elevated risk, with an OR of 1.33 (95% CI: (1.16, 1.57), *p* < 0.001), indicating a 33% increase in odds of poor outcomes per 0.1-unit increment. In contrast, when SHR exceeds 1.09, the association becomes non-significant, with an OR of 0.91 (95% CI: 0.79–1.06, *p* = 0.201) per 0.1-unit increase, indicating that the detrimental effect of stress hyperglycemia on functional outcomes is predominantly observed below this threshold, suggesting a trend toward reduced risk, though not statistically significant. The log-likelihood ratio test showed a *p*-value of 0.002, confirming a significant model fit and a nonlinear relationship between SHR and functional outcomes. These findings suggest that SHR may serve as a prognostic indicator warranting further investigation, and the identified threshold effect provides a hypothesis for future prospective studies examining glycemic management strategies in acute ischemic stroke.

### Results of subgroup analyses

In all prespecified or exploratory subgroup assessments (Table 6), age, sex, smoking status, diabetes, and stroke/TIA did not significantly interact with SHR. These influences did not modify or alter the relationship between SHR and 90-day functional outcome in patients with AIS.

**Table 6:** Stratified associations between SHR and 90-day functional outcome in patients with AIS by age, sex, hypertension, smoking status, diabetes, and stroke/TIA.

Characteristic	No. of patients	Effect size (95%CI)	*p*-value	*p* for interaction
Sex				0.516
Male	928	2.01 (0.78, 5.19)	0.147	
Female	393	4.61 (1.27, 16.78)	0.021	
Age (years)				0.193
<65	889	1.15 (0.37, 3.58)	0.804	
≥65	432	8.40(2.57, 27.47)	<0.001	
Smoking				0.747
Current	368	1.54 (0.24, 9.81)	0.645	
Ever	100	1.33 (0.05, 37.73)	0.868	
Never	853	3.13 (1.29, 7.62)	0.012	
Diabetes				0.308
No	1,095	3.43 (1.48, 7.98)	0.004	
Yes	226	2.71 (0.38, 19.38)	0.320	
Stroke				0.942
No	1,096	3.01 (1.2, 6.84)	0.009	
Yes	225	3.34 (0.37, 30.48)	0.285	

The above model adjusted for sex, age, education, smoking, BMI, diabetes, stroke/TIA, atrial fibrillation, dyslipidemia, SBP, Hs-CRP, DNT, cystatin C, TOAST, NIHSS, pre-mRS, antiplatelet therapy, and anticoagulant therapy.

In each case, the model is not adjusted for the stratification variable.

OR, odds ratios; CI, confidence.

## Discussion

In this retrospective cohort study involving 1,321 consecutive patients with acute ischemic stroke, we investigated the association between stress hyperglycemia ratio (SHR) and 90-day functional outcome. After comprehensive adjustment for demographic characteristics, cardiovascular risk factors, and clinical parameters, we identified a significant non-linear relationship between SHR and poor outcome. Notably, for SHR values below 1.09, each 0.1-unit increase in SHR was associated with substantially elevated risk of poor outcome (OR = 1.33, 95%CI: (1.16, 1.57), *p* < 0.001). This association significantly attenuated beyond the threshold of 1.09, where each 0.1-unit increase showed no significant association (OR = 0.91, 95%CI: 0.79–1.06, *p* = 0.201), suggesting a clear prognostic threshold effect of SHR.

SHR plays a crucial role in reflecting acute glycemic response during physiological stress, independent of chronic glycemic status. It serves as a significant predictor of adverse clinical outcomes and provides valuable prognostic information in critically ill patients (21, 27). Our findings align with several recent studies. Shen et al. (17) demonstrated a significant association between SHR and clinical outcomes in a study of 341 acute ischemic stroke patients receiving intravenous thrombolysis. Similarly, Liu et al.’s (5) study (*n* = 819) confirmed the relationship between SHR and stroke prognosis. However, these studies primarily employed conventional linear regression models, failing to identify the threshold effect we discovered. Our study, through piecewise linear regression analysis, was the first to establish a critical prognostic threshold of SHR at 1.09, providing a more precise reference for clinical risk stratification. A recent multicenter study by Huang et al. suggested that SHR might influence stroke outcomes through its effects on inflammatory responses and oxidative stress (28). Interestingly, while our research identified a specific inflection point, some recent studies have suggested that SHR may have a U-shaped relationship with certain clinical outcomes. This potential non-linear pattern warrants further investigation to determine whether our identified threshold represents one component of a more complex relationship between SHR and stroke outcomes (27). Regarding potential mechanisms, multiple studies have confirmed that SHR might influence stroke outcomes through its effects on inflammatory responses and oxidative stress, with stress hyperglycemia being associated with enhanced inflammatory responses and elevated oxidative stress levels, which may be important mechanisms by which SHR affects stroke prognosis (29). Furthermore, our study featured more comprehensive confounder adjustment compared to previous research, including important covariates such as education level, medical history, and laboratory parameters, enhancing the reliability of our findings.

To contextualize the magnitude of our findings, we note that a 0.1-unit increase in SHR (for example, from 0.95 to 1.05) was associated with a 33% increase in the odds of poor functional outcomes when SHR remained below the threshold of 1.09 in our cohort. This association provides a basis for quantifying the relationship between stress hyperglycemia and functional prognosis in this population. However, we emphasize that these are observational associations from a retrospective study and should not be directly applied as clinical decision-making thresholds. The cumulative relationship implied by the piecewise model (OR_cumulative = 1.33^*n* for *n* 0.1-unit increments below SHR = 1.09) provides a statistical characterization of the dose–response pattern in our dataset, and may inform the design of future prospective studies or interventional trials examining glycemic management in acute ischemic stroke. Formal clinical application of any SHR-based risk stratification approach would require prospective validation in independent cohorts, assessment of clinical utility beyond prognostic association (including net reclassification improvement and decision curve analysis), and demonstration of benefit from SHR-guided interventions in randomized trials. An important methodological consideration in our study is that admission glucose measurements were obtained as random (non-fasting) samples in the emergency setting. We argue that this approach is not only practically necessary but also physiologically appropriate in the acute stroke context for the following reasons: First, in acute ischemic stroke, the dominant driver of elevated admission glucose is the neuroendocrine stress response: activation of the HPA axis and sympathoadrenal system leads to catecholamine and cortisol release, promoting hepatic gluconeogenesis, glycogenolysis, and peripheral insulin resistance (30, 31). This stress-mediated glucose elevation (typically 5–10 mmol/L above individual baseline) substantially exceeds normal postprandial fluctuations (2–3 mmol/L in non-diabetic individuals), and occurs irrespective of fasting status. Therefore, admission glucose in the acute stroke setting predominantly reflects pathological stress hyperglycemia rather than nutritional state. Second, the SHR metric’s design normalizes acute random glucose against HbA1c-derived estimated average glucose, which reflects an individual’s glycemic levels across both fasting and postprandial states over the preceding 2–3 months—thereby providing a personalized, fasting-status-independent baseline adjustment. This design inherently mitigates the impact of fasting status variability on SHR. Third, the use of random admission glucose for SHR calculation is well-established and consistent with prior published SHR studies in acute ischemic stroke. Notably, Chen *et al.* (16) directly compared random versus fasting glucose-based SHR in IVT-treated AIS patients, demonstrating that the random glucose-based SHR (SHR1) was a significant independent predictor of 90-day functional outcomes in non-diabetic patients (OR = 1.246, 95% CI: 1.041–1.492, *p* = 0.016). Multiple subsequent studies employing the same approach have reported consistent prognostic associations (10, 15, 17), corroborating the robustness of the SHR–outcome relationship when using random admission glucose. Fourth, the pronounced threshold effect identified in our study (OR = 1.33 per 0.1-unit for SHR < 1.09) is entirely consistent with a stress-related physiological mechanism, and would be implausible if random glucose variability were the dominant signal. We acknowledge, however, that as a retrospective study, meal timing and time-of-day of glucose measurement were not systematically documented, precluding direct quantification of this potential source of variability. Future prospective studies with systematic documentation of meal timing and sampling time would provide more definitive evidence. We have added this as an explicit limitation in the Limitations section. Specifically, future prospective studies should formally assess the incremental predictive value of SHR beyond established predictors—including NIHSS score, age, and stroke subtype—through net reclassification improvement (NRI) and integrated discrimination improvement (IDI) analyses, which are necessary to establish whether SHR provides clinically meaningful prognostic information above and beyond current risk stratification tools. Our findings have potential implications for future prognostic research. First, the identified SHR threshold (1.09) represents an association-based prognostic signal that, if validated prospectively, could potentially inform risk stratification approaches in acute ischemic stroke patients receiving intravenous thrombolysis. However, we emphasize that our retrospective findings should not be interpreted as establishing a clinical action threshold; prospective validation is required before any SHR-based criterion could be considered for clinical use. Second, the non-linear association we observed raises hypotheses regarding glycemic management strategies that warrant investigation in future interventional studies: specifically, whether patients with SHR below 1.09 might benefit from enhanced glycemic monitoring, and whether the risk–benefit profile of glucose-lowering therapy differs across the SHR spectrum. These questions can only be definitively answered through prospective randomized trials. Furthermore, these findings contribute exploratory observational evidence that may help prioritize future clinical research directions, but do not constitute evidence-based support for clinical practice guidelines in their current form. However, large-scale multicenter randomized controlled trials are still needed to validate these findings, particularly regarding the applicability of the SHR threshold across different ethnic groups and stroke subtypes, as well as the effectiveness of clinical interventions based on this threshold.

Several strengths of our study merit emphasis. First, our retrospective multicenter design, underpinned by the centralized governance structure of the Shenzhen Municipal Stroke Quality Control Center, substantially enhanced the reliability and generalizability of our findings. The unified web-based data entry platform, standardized personnel training, centralized data auditing, and geographic concentration of all 36 centers within a single metropolitan healthcare network collectively provided robust cross-center data standardization—a level of quality control exceeding that typically achievable in retrospective registries spanning heterogeneous healthcare systems. Second, we employed comprehensive statistical approaches, including both conventional regression analyses and advanced threshold effect modeling, to provide deeper insights into the complex relationship between SHR and stroke outcomes. Third, our analysis accounted for an extensive range of potential confounders across demographic, clinical, and laboratory domains (Model III included sex, age, education, smoking, BMI, diabetes, stroke/TIA history, atrial fibrillation, dyslipidemia, systolic blood pressure, Hs-CRP, door-to-needle time, cystatin C, TOAST classification, NIHSS score, pre-stroke mRS, antiplatelet therapy, and anticoagulant therapy). Critically, we conducted a pre-specified *E*-value sensitivity analysis ((26)) to quantitatively assess robustness to potential unmeasured confounding. The *E*-value for the primary association (*E*-value = 5.17; lower CI: 2.06) and for the threshold effect below SHR = 1.09 (*E*-value = 44.26; lower CI: 9.83) both substantially exceed the strength of association attributable to any plausible unmeasured confounder—including glucose-lowering medications (typical RR: 1.1–1.7), dietary habits, or genetic variations—thereby providing quantitative evidence that our findings are robust to residual unmeasured confounding. Fourth, the 90-day functional outcomes were assessed through standardized structured telephone interviews conducted by trained neurologists as part of routine registry data collection, independently of and prior to the investigators’ analysis of SHR-outcome associations. This procedural independence between outcome ascertainment and exposure analysis minimizes the risk of assessor bias inherent to retrospective observational studies. Furthermore, the telephone mRS assessment followed a validated structured interview protocol (18, 19), ensuring consistency and reliability of outcome measurement across all participating centers. Finally, our large sample size of consecutive patients receiving intravenous thrombolysis provided sufficient statistical power to detect clinically meaningful associations and enabled robust subgroup analyses.

Several limitations should be acknowledged. First, our database did not systematically capture information on endovascular thrombectomy (EVT) performed following intravenous thrombolysis. This limitation prevents us from determining whether any patients received interventional surgery, analyzing the effect of thrombectomy on patient prognosis, or assessing whether the SHR-outcome relationship differs between IVT-only and bridging therapy patients. However, during our study period (2017–2022), EVT utilization rates were relatively low in our region, and we adjusted for baseline NIHSS score as a proxy for stroke severity. Future prospective studies with comprehensive EVT data collection are needed to validate whether the identified SHR threshold remains consistent across different reperfusion strategies. Second, although admission blood glucose was a random (non-fasting) measurement—which is standard and physiologically appropriate in the acute stroke emergency setting—meal timing and time-of-day of glucose sampling were not systematically documented in our retrospective database, precluding direct quantification of nutritional contribution to glucose variability. This limitation is mitigated by multiple factors: (1) the SHR design normalizes random glucose against HbA1c-derived average glucose reflecting both fasting and postprandial states over the preceding 2–3 months; (2) the predominance of stress-mediated glucose elevation (5–10 mmol/L) over typical postprandial fluctuation (2–3 mmol/L) in acute stroke; and (3) the consistency of our findings with prior SHR studies using the same measurement approach, including a study directly comparing random versus fasting glucose-based SHR (16). Prospective studies with systematic meal timing documentation would more definitively characterize this source of variability. Third, we excluded patients with pre-existing diabetes, severe organ dysfunction, and those receiving glucose-lowering medications, potentially limiting the generalizability of our findings to these populations. Fourth, as an observational study, we could only demonstrate associations rather than establish causality between SHR and functional outcomes. Fifth, while we adjusted for an extensive set of measurable confounders (Model III), several important variables were unavailable in our retrospective database and represent potential unmeasured confounders: (1) pre-stroke glucose-lowering medication use—types and dosages of antidiabetic medications (metformin, sulfonylureas, DPP-4 inhibitors, GLP-1 receptor agonists, SGLT-2 inhibitors, and insulin) were not systematically collected across all 36 centers, as medication reconciliation was not standardized in the retrospective database; certain agents with reported pleiotropic neuroprotective effects (e.g., GLP-1 receptor agonists and SGLT-2 inhibitors) could theoretically influence both SHR levels and functional outcomes; (2) infarct volume and recanalization status—neuroimaging quantification and systematic recanalization data were not uniformly available across centers; (3) dietary habits, physical activity, and lifestyle factors; and (4) pharmacogenomic and glycemic regulation-related genetic variants. However, the *E*-value analysis provides strong quantitative evidence that these unmeasured confounders are unlikely to fully explain our findings: an unmeasured confounder would need to be associated with both SHR and poor outcomes by a risk ratio of at least 5.17-fold (primary association) or 44.26-fold (threshold effect below SHR = 1.09)—far exceeding the effect size of any plausible unmeasured confounder reported in the literature. Future prospective studies with systematic collection of pre-stroke medication histories and comprehensive neuroimaging data will be critical for definitively addressing these residual confounders. Sixth, our study population consisted predominantly of Chinese patients from eastern China, potentially limiting the applicability of our findings to other ethnic groups. Finally, we only assessed functional outcomes at 90 days post-stroke, and the long-term impact of SHR on stroke outcomes remains to be determined. Furthermore, while the centralized governance of the Shenzhen Municipal Stroke Quality Control Center and unified data entry protocols substantially mitigated inter-center variability, we did not formally account for center-level clustering effects in our primary analyses through mixed-effects models or generalized estimating equations (GEEs). As a retrospective study, unmeasured inter-center variations in thrombolysis agent selection, imaging eligibility criteria, post-stroke glycemic management, and post-discharge follow-up intensity may have introduced residual center-level heterogeneity that our covariate adjustment cannot fully address. Future prospective studies should pre-specify hierarchical modeling approaches (e.g., mixed-effects logistic regression with center as a random effect) as the primary analysis strategy, and systematically collect center-level characteristics as covariates, to formally evaluate and account for center-level clustering effects on the SHR-outcome relationship. Our findings highlight several important directions for future investigation. Prospective multicenter studies with systematic collection of comprehensive endovascular thrombectomy data are urgently needed to validate whether the SHR threshold effect (inflection point at 1.09) remains consistent across different reperfusion strategies, including IVT alone, bridging therapy (IVT + EVT), and direct EVT approaches, and to assess whether successful recanalization (TICI 2b-3) modifies the prognostic value of stress hyperglycemia and whether the timing of recanalization interacts with acute glycemic stress to influence outcomes. Mechanistic studies are warranted to elucidate the biological pathways through which acute glycemic stress differentially influences outcomes above and below the identified threshold, with potential mechanisms including threshold-dependent effects on oxidative stress, neuroinflammation, blood–brain barrier integrity, hemorrhagic transformation risk, and reperfusion injury, which could identify novel therapeutic targets for neuroprotection in hyperglycemic stroke patients. Interventional trials are needed to determine whether intensive glycemic management strategies targeting SHR below the 1.09 threshold can improve functional outcomes, and whether such interventions should be tailored based on reperfusion therapy type, stroke severity, or recanalization status, with randomized controlled trials comparing different glycemic management approaches (intensive vs. conventional) in well-defined patient subgroups providing critical evidence for clinical practice. External validation studies in diverse populations, healthcare systems, and geographic regions are essential to confirm the generalizability of our findings and establish whether the SHR threshold varies across different ethnicities, baseline glycemic control status (pre-diabetic vs. non-diabetic), stroke subtypes (large vessel atherosclerosis vs. cardioembolic), or healthcare resource settings (high vs. low EVT availability). Additionally, future prospective multicenter studies should adopt pre-specified hierarchical modeling strategies (mixed-effects logistic regression with center as a random effect, or GEE with center as a clustering variable) as the primary analytical framework, building upon the existing Shenzhen Stroke Center Database infrastructure to formally characterize and adjust for center-level clustering effects. Systematic collection of center-level variables—including annual thrombolysis volume, stroke unit certification status, glycemic management protocols, and EVT capability—will enable formal evaluation of center-level effect modification and provide more definitive evidence for the generalizability of the SHR threshold across diverse clinical settings. The standalone discriminatory performance of SHR (AUC = 0.573) is modest, consistent with prior literature on single glycemic biomarkers in multifactorial stroke outcome prediction. This finding reinforces that SHR is intended as a complementary prognostic indicator alongside established predictors (NIHSS, age, stroke subtype) rather than a standalone predictive model. Future prospective studies should incorporate formal calibration assessment (e.g., Hosmer–Lemeshow test and calibration plots) within fully adjusted multivariable models to provide a comprehensive evaluation of model performance.

While our study provides robust evidence for the independent prognostic value of SHR in acute ischemic stroke, several important questions remain for future investigation. First, prospective studies with systematic, standardized collection of pre-stroke medication histories are warranted to definitively examine the potential modifying effects of specific glucose-lowering drug classes. Such studies should capture detailed information on medication types (metformin, sulfonylureas, DPP-4 inhibitors, GLP-1 receptor agonists, SGLT-2 inhibitors, and insulin), dosages, treatment duration, and adherence patterns. This would allow investigators to determine whether certain medications with reported pleiotropic effects (e.g., SGLT-2 inhibitors and GLP-1 agonists) modify the SHR-outcome relationship or provide additional prognostic information beyond SHR. Second, mechanistic studies are needed to elucidate the pathophysiological pathways linking stress hyperglycemia to poor outcomes.

## Conclusion

Our multicenter retrospective study identified a significant non-linear association between SHR and 90-day functional outcomes in acute ischemic stroke patients receiving intravenous thrombolysis, with an inflection point at SHR = 1.09. Below this threshold, each 0.1-unit increase in SHR was associated with a 33% increased odds of poor functional outcomes (OR = 1.33, 95% CI: 1.16–1.57, *p* < 0.001), an association robust to unmeasured confounding (*E*-value = 44.26). These findings suggest SHR holds promise as a prognostic biomarker derivable from routine admission data; however, prospective validation in diverse cohorts, formal assessment of incremental predictive value beyond established predictors (NIHSS, age, and stroke subtype) through NRI and IDI analyses, and demonstration of benefit in randomized controlled trials are necessary before SHR can inform thrombolysis decisions. Future prospective multicenter studies should incorporate comprehensive reperfusion data (EVT and recanalization status), neuroimaging parameters, and longer-term outcomes to fully characterize the SHR-outcome relationship across contemporary acute stroke care. While our study could not assess patients receiving endovascular thrombectomy due to systematic data collection limitations inherent to the retrospective design, our findings provide valuable insights for risk stratification and prognostic assessment in IVT-treated patients, who represent the majority of reperfusion therapy recipients globally. Future prospective studies incorporating comprehensive data on all reperfusion modalities, including EVT procedures and recanalization status, are essential to fully characterize the SHR-outcome relationship across the complete spectrum of contemporary acute stroke treatments and to determine whether the identified threshold effect is modified by mechanical thrombectomy and successful vessel recanalization.

## Data Availability

The raw data supporting the conclusions of this article will be made available by the authors, without undue reservation.

## Footnotes

1
http://www.R-project.org
