# Supplementary figures and images for: Impacts of stress hyperglycemia ratio on functional outcomes of ischemic stroke patients treated with intravenous thrombolysis: a population-based study
Source: Front Neurol. 2026 Mar 23;17:1685472. doi: 10.3389/fneur.2026.1685472 (PMC13050741; doi:10.3389/fneur.2026.1685472)

**SHR**  
**AUC = 0.573**

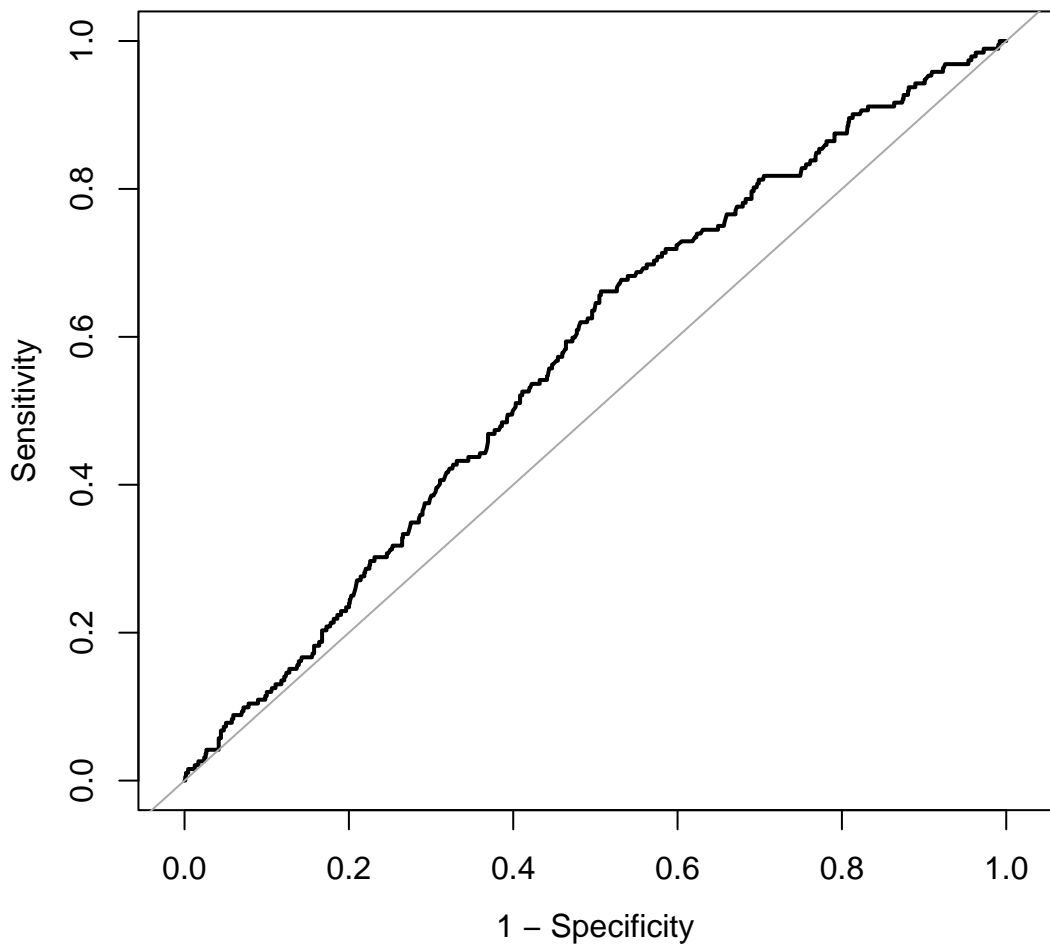

Supplement: Supplementary file 1 [file Data_Sheet_1.PDF]

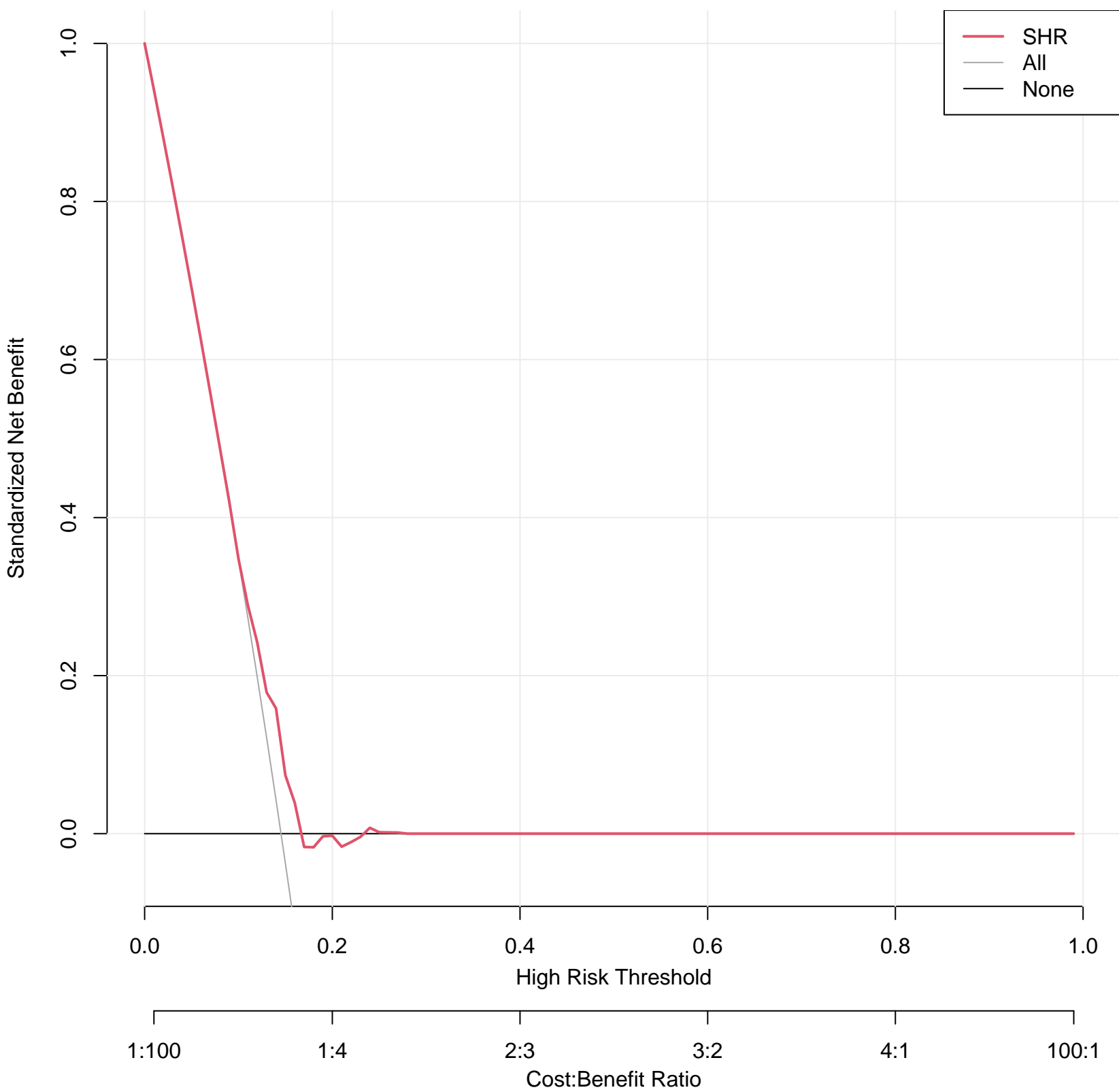

Supplement: Supplementary file 2 [file Data_Sheet_2.PDF]
